# Case Report: Omalizumab for Chronic Spontaneous Urticaria in Pregnancy

**DOI:** 10.3389/fimmu.2021.652973

**Published:** 2021-03-16

**Authors:** Shuang-Lu Liao, Miao Yu, Zuo-Tao Zhao, Marcus Maurer

**Affiliations:** ^1^ Department of Dermatology and Venerology, Peking University First Hospital, Beijing, China; ^2^ Beijing Key Laboratory of Molecular Diagnosis on Dermatoses, Beijing, China; ^3^ National Clinical Research Center for Skin and Immune Diseases, Beijing, China; ^4^ Peking University School of Nursing, Beijing, China; ^5^ Dermatological Allergology, Allergie-Centrum-Charité, Department of Dermatology and Allergy, Charité - Universitätsmedizin Berlin, Berlin, Germany

**Keywords:** omalizumab, pregnancy, chronic spontaneous urticaria, wheals, angioedema

## Abstract

Most chronic spontaneous urticaria (CSU) patients are female, and pregnancy can aggravate the disease activity of patients, but little is known about the efficacy and safety of omalizumab in pregnant CSU patients. We report two pregnant CSU patients treated with omalizumab and review the published information on omalizumab treatment during 11 pregnancies. The outcomes reported on patients with known pregnancies showed they had normal pregnancies and healthy babies as well as complete control of their CSU. The two new cases we reported support the view that omalizumab could be an effective and safe treatment option for pregnant and breastfeeding CSU patients. Further high-quality studies need to be carried out in order to obtain more information on the long-term efficacy and safety of the use of omalizumab during pregnancy in patients with chronic urticaria, including CSU.

## Introduction

Chronic spontaneous urticaria (CSU) is a heterogeneous disorder with recurrent pruritic wheals and angioedema or both that markedly affects patients’ quality of life (1, 2). The anti-IgE antibody omalizumab is used in CSU patients resistant to antihistamine treatment (3). Most CSU patients are female, and little is known about the efficacy and safety of omalizumab in pregnant CSU patients. We treated patient #1 and patient #2 at Peking University First Hospital with omalizumab and reviewed the published information on this treatment during pregnancy.

## Case Description

The first patient is a 33-year-old woman diagnosed with CSU and comorbid symptomatic dermographism 2 years ago. She had a history of spontaneously occurring recurrent pruritic wheals, most often on the extremities and trunk, that would last less than 24 h. As confirmed by provocation testing, patient #1 also developed wheals in response to rubbing of skin that happened in real life, and this was true even with minor triggers such as drying herself with a towel after showering. Patient #1 had not experienced angioedema. Treatment with first cetirizine 10mg/day and then, due to drowsiness, ebastine 10mg/day for several weeks did not reduce disease activity. As Patient #1 did not consent to the use of a higher than standard dosed antihistamine, we initiated treatment with omalizumab, in June 2020, at 300mg/month, which led to complete control as assessed by the use of the urticaria control test (UCT). The UCT assesses disease control in patients with CSU with four questions, each with five answer options (scored with 0–4 points), where a low total score indicates poor disease control and the maximum total score of 16 reflects complete control (4, 5). The UCT score of patient #1, 4 weeks after starting omalizumab, was 16. During the first 4 weeks, patient #1 also experienced a marked improvement of quality of life (QoL) impairment as assessed by the Dermatology Life Quality Index (DLQI) and the Chronic Urticaria QoL Questionnaire (CU-Q_2_oL). For the DLQI and CU-Q_2_oL, higher scores reflect higher QoL impairment, and a score of 0–1, for the DLQI, and of 23, for the CU-Q_2_ol, indicated that there was no QoL impairment. A total of 4 weeks after the start of omalizumab application, the DLQI and CU-Q_2_oL scores were 0 and 23, respectively. After 10 days of the third omalizumab application (August 2020), 10 weeks after the first application, patient #1 was found to be 10 weeks pregnant. She chose to stop omalizumab, continued with cetirizine treatment, 10 mg every 3 days, and has remained free of CU signs and symptoms since then.

The second patient is a 36-year-old woman with CSU. She had recurrent generalized wheals with pruritus (daily or almost daily for 5 years) that lasted for several hours each time and occurred without known triggers. Serum total IgE concentration was measured by a chemiluminescent immunoassay (ImmunoCAP; Thermo-Fisher Scientific, Sweden), and levels of 100 kU/L or greater were defined as increased. Thyroid autoantibodies, including serum anti-thyroid peroxidase antibody (anti-TPO IgG) and anti-thyroglobulin antibody (anti-TG IgG), were determined using an electrochemiluminescence immunoassay (Roche Elecsys-2010; Roche Diagnostics, U.S.) with normal reference ranges of 0–34 IU/ml and 0–115 IU/ml, respectively; the serum total IgE of Patient #2 was low at 19.3 kU/L, anti-TPO IgG was found to be elevated at 87 IU/ml, and anti-TG IgG was normal at 20 IU/ml). A biopsy taken from lesional skin of patient #2 showed a perivascular inflammatory infiltrate of lymphocytes and eosinophils, erythrocyte extravasation, and scant edema within the superficial and mid dermis, without fibrinoid deposits and leukocytoclasis, consistent with urticaria. Patient #2, after being diagnosed with CSU in June 2018, was treated with cyclosporine and was controlled well during the 10 months of treatment. In May 2019, CSU showed exacerbation, cyclosporine was stopped, and omalizumab treatment was started (UCT:1; DLQI:15; CU-Q2ol:80). Three days after the first application (300 mg), she was free of CSU signs and symptoms but only for 5 days. We increased the dose of a second application after 4 weeks to 450 mg, which led to complete recovery within 3 days for 3 months, at 450 mg/month (UCT:16; DLQI:0; CU-Q2ol:23). We then decreased the dose to 300 mg/month, and complete control was maintained by this treatment until January 2020, when patient #2 experienced severe exacerbation of CSU phenotype (UCT:1; DLQI:12; Cu-Q2ol:65) and was found to be 12 weeks pregnant. With the patient’s informed consent, we increased the dose of omalizumab to 450 mg, and she regained complete control within 3 days. Two attempts to reduce to 300 mg/month failed, and 450 mg/month omalizumab treatment was maintained until August 2020, when patient #2 gave birth to a full-term healthy male infant (weight 3350g, length 50cm). Patient #2 is breastfeeding her child and is symptom-free with 450 mg/month omalizumab.

Patient #1 and Patient #2 have given written informed consent to the publication of their case details. The study was conducted according to the Declaration of Helsinki. It was approved by the Chinese Ethics Committee of Registering Clinical Trial (ChiECRCT20190131) and registered with the Chinese clinical trial registry (ChiCTR1900024869).

## Discussion

Taken together, both patients described herein became pregnant while on omalizumab treatment, both achieved complete response during pregnancy (albeit at higher than the standard dose in one patient), and both pregnancies were unproblematic.

At this time, only 11 pregnancies in CSU patients treated with omalizumab were reported in the literature (**Table 1** (6–11)). Similar to patients #1 and #2, almost all patients previously reported had normal pregnancies and healthy babies as well as complete control of their CSU. Of note, increasing the dose of omalizumab during pregnancy was only reported in one previous CSU patient. In this patient, the first CSU patient ever reported to receive omalizumab during pregnancy, disease activity while on omalizumab 150mg/month increased markedly, and intervals were shortened to every 15 days, which resulted in complete remission (11). Patient #2 was also given an increased dose during pregnancy, from 300mg to 450mg omalizumab per month, and is the first pregnant CSU patient reported to receive more than the licensed dosed omalizumab. Nonresponse to standard dosed omalizumab in CSU had not previously been linked to high body weight, and patient #2 was 70–80 kg (before, during, and after pregnancy) and 165 cm, i.e., not overweight. Omalizumab blood concentrations of patient #2 had not been assessed as no assay to do so was available to us and nonresponse to standard dosed omalizumab in CSU had not previously been linked to altered blood levels of omalizumab.

**Table 1:** Characteristics of reported cases of pregnant CU patients with omalizumab treatment.

	Title	Author	year	disease	number of patients	Age range	Timing of exposure	Omalizumab dose/interval	Evidence of efficacy	Evidence for safety								
										Maternal adverse events	Live births	Small for Gestational Age	Low birth weight	Preterm birth(<37wk)	Stillbirth/fetal death(≥20wk)	Spontaneous abortion(<20wk)	congenital malformations	neonatal adverse outcomes
1	Our study	Our group	2020	CU	2	33,37	First Trimester	1/2:150-300mg/4 wk, 1/2:300-450mg/4 wk	complete control	0/2	1/1^†^	0/1	0/1	0/1	0/1	0/1	0/1	0/1
2	Omalizumab concentrations in pregnancy and lactation: A case study	Saito, J. et al.	2020	CSU	1	38	First Trimester	150mg/4 wk	complete control	0/1	1/1	0/1	0/1	0/1	0/1	0/1	skin defect, aortic aneurysm	0/1
3	Omalizumab as Third-Line Therapy for Urticaria During Pregnancy	Ensina, L. F. et al.	2017	CSU	2	29,32	First Trimester	1/2:150 mg/4 wk, 1/2:300mg twice with an interval of 12 wk	complete control	0/2	3/3	0/3	0/3	0/3	0/3	0/3	0/3	0/3
4	Omalizumab use during pregnancy for chronic spontaneous urticaria (CSU): report of two cases	González-Medina, M. et al.	2017	CSU	2	37,37	First Trimester	300 mg/4 wk	complete control	0/2	2/2	0/2	0/2	0/2	0/2	0/2	0/2	0/2
5	Omalizumab use during pregnancy for CIU: a tertiary care experience	Cuervo-Pardo L. et al.	2016	CSU	4	25-28	First Trimester	300 mg/4 wk	complete control	0/4	4/4	0/4	0/4	0/4	0/4	0/4	0/4	0/4
6	Successful and Safe Treatment of Chronic Spontaneous Urticaria with Omalizumab in a Woman during Two Consecutive Pregnancies	Ghazanfar, M. N. et.al.	2015	CSU	1	32	First Trimester	150mg/2wk, 300mg/4wk	complete control	0/1	2/2	0/2	0/2	0/2	0/2	0/2	0/2	0/2
7	Effects of omalizumab in a patient with three types of chronic urticaria	Vieira Dos Santos R. et al.	2014	CSU CIndU	1	37	First Trimester	150mg/4wk, 150mg/2wk	complete control	0/1	1/1^†^	0/1	0/1	0/1	0/1	0/1	0/1	0/1

†The other patient was still pregnant. CU, Chronic Urticaria; CSU, Chronic Spontaneous Urticaria; CIndU, inducible urticaria.

No evidence of a relationship between omalizumab exposure and increased risk of adverse events in pregnant patients and their infants was reported (12). Omalizumab can be an effective option for pregnant CSU patients and those who want to become pregnant.

## Data Availability Statement

The raw data supporting the conclusions of this article will be made available by the authors, without undue reservation.

## Ethics Statement

Ethical review and approval was not required for the study on human participants in accordance with the local legislation and institutional requirements. The patients/participants provided their written informed consent to participate in this study. Written informed consent was obtained from the individual(s) for the publication of any potentially identifiable images or data included in this article.

## Author Contributions

S-LL: performed data analysis and drafted the manuscript. MY: performed data analysis and prepared the manuscript. Z-TZ: designed the study and prepared the manuscript, reviewed the article critically for important intellectual content. MM: drafted the manuscript, reviewed the article critically for important intellectual content. All authors contributed to the article and approved the submitted version.

## Conflict of Interest

The authors declare that the research was conducted in the absence of any commercial or financial relationships that could be construed as a potential conflict of interest.

## Abbreviations

CSU, Chronic Spontaneous Urticaria; UCT, Urticaria Control Test; DLQI, Dermatology Life Quality Index; CU-Q2ol, Chronic Urticaria Quality of Life Questionnaire.
